# A Clean and Facile Synthesis Strategy of MoS_2_ Nanosheets Grown on Multi-Wall CNTs for Enhanced Hydrogen Evolution Reaction Performance

**DOI:** 10.1038/s41598-017-09047-x

**Published:** 2017-08-18

**Authors:** Jiamu Cao, Jing Zhou, Yufeng Zhang, Xiaowei Liu

**Affiliations:** 1 MEMS Center, Harbin Institute of Technology, Harbin, 150001 China; 2 Key Laboratory of Microsystems and Microstructures Manufacturing, Ministry of Education, Harbin, 150001 China

## Abstract

Unique hybrid nanostructure, which consists of multi-wall carbon nanotube (MWCNT) stems and MoS_2_ nanosheet (NS) leaves, are prepared by a hydrothermal method. The fabricated material can be potentially used as an electrocatalyst for the hydrogen evolution reaction (HER). To our knowledge, as the reaction medium, water is firstly utilized to the synthesis of the 1T phase MoS_2_ NSs which uniformly grow on the carbon-based materials. As a result, a nanohybrid catalyst with excellent HER electrocatalytic properties, which included an onset potential of as low as 50 mV, a Tafel slope of 43 mV dec^−1^, and remarkable cycling stability, is produced. The observed outstanding catalytic performance can be attributed to the uniform distribution of the metallic 1T phase of the MoS_2_ NSs, which are characterized by the presence of multiple active edges as well as the effective electron transport route provided by the conductive MWCNT substrate. This work demonstrates the high potential of the synthesized HER catalyst and proposes a novel, efficient, environmentally friendly, and inexpensive method for its fabrication.

## Introduction

Hydrogen gas is used as a scalable and renewable energy source, which can be produced via an environmentally clean conversion chain (from the production to the utilization stages)^1^. Owing to these advantages, hydrogen can become a promising source of alternative energy, which would solve the existing environmental emission issues related to the use of fossil fuels^2^. Due to its sustainable characteristics, water splitting has become one of the most widely considered approaches for hydrogen production^3^. During the hydrogen evolution reaction (HER), the utilized electrocatalyst reduces the overpotential of electrodes to a very low value that matches the incoming solar photon flux, which in turn produces high current density and consequently increases the yield of the electrochemical process^4^. Until today, metals of the Pt-group have been widely utilized as the most effective HER catalysts^5, 6^. However, their high material costs and limited resources make the use of hydrogen for energy production less feasible^7–9^. As a result, the demand for an inexpensive alternative HER catalyst remains relatively high^10–13^.

MoS_2_ is a typical transition metal sulfide with a layered structure held together by weak van der Waals forces, which represents an abundant, geographically ubiquitous, and potentially cheap analog of graphene^14, 15^. The recent computational and experimental studies revealed that MoS_2_ could be used as a competitive HER electrocatalyst containing active edge sites, which attracted significant interest from various researchers working in the field of water splitting^16–20^. However, the poor intrinsic conductivity of MoS_2_ negatively affects its charge transport properties^21, 22^. To mitigate this issue, MoS_2_-based catalysts must be grafted onto the surface of conductive substrates using polymer binders as film-forming agents^23^. During this procedure, some active sites become blocked causing the deterioration of the catalytic properties of MoS_2_^24^. Therefore, the development of nanosized MoS_2_ particles, which are directly supported on a highly conductive substrate with a large surface area, is critical for the enhancement of their electrocatalytic efficiency in practical applications^25^.

Multi-wall carbon nanotubes (MWCNTs) are one-dimensional materials, which possess high electrical conductivity, excellent chemical stability, and large surface area (they are also 20 times cheaper than graphene)^26, 27^. Owing to these advantages, MWCNTs can be used as one of the most promising supports for nano-sized catalysts. In previous reports, the hybrid of nano-sized MoS_2_/carbon-based material was prepared in the dimethylformamide (DMF) solution, while replacing the not environmental-friendly DMF with water only leads to a simple mixture of nano-sized MoS_2_ particles and carbon sources^28–37^. In this study, a highly effective electrocatalyst for HER which petal-like metallic 1T phase MoS_2_ nanosheets (NSs) hydrothermally uniformly grown on the MWCNT surface was successfully synthesized in aqueous solution for the first time.

## Methods

### Surface functionalization of MWCNTs

MWCNTs with average outer/inner diameters of 15 nm/8 nm and lengths of 50 μm were purchased from XF Nano, Ltd (Nanjing, CN). To improve the hydrophilicity of the nanotube substrate, the MWCNT surface was functionalized prior to MoS_2_ deposition. First, 100 mg of MWCNTs was sonicated in 500 mL of concentrated HNO_3_ solution (70% w/w) at a temperature of 60 °C for 1 h. After that, the MWCNT sample was washed with deionized (DI) water and dried at 60 °C in a vacuum oven for 6 h.

### Preparation of MoS_2_ NS/MWCNT hybrid catalyst

During the synthesis of the MoS_2_ NS/MWCNT hybrid material, 56 mg of sodium molybdate, 67 mg of thiourea, and 11 mg of surface-functionalized MWCNTs were added to 70 mL of DI water and sonicated for 1 h. Then, the hybrid was transferred to a 100 mL Teflon-lined autoclave and heated at 180 °C for 24 h. The resulting dark suspension was collected via centrifugation at a speed of 6000 rpm, and then washed with DI water and ethanol, and dried in the vacuum oven at 60 °C. For comparison, MoS_2_ nanoflowers (NFs) were synthesized via a similar method without MWCNT addition.

### Material characterization

The morphology and structure of the produced nanohybrid material were characterized using a scanning electron microscope (SEM, Helios Nanolab-600) operated at an accelerating voltage of 200 kV, which was equipped with an energy dispersive spectroscopic (EDS) detector; a transmission electron microscope (TEM, Tecnai F2 F30); and a high-resolution transmission electron microscope (HRTEM) operated at an acceleration voltage of 200 kV. Furthermore, the chemical composition and atomic valence states of the MoS_2_ NS/MWCNT composite were investigated via X-ray photoelectron spectroscopy (XPS, Thermo Fisher Scientific Company K-Alpha). Raman spectroscopy was performed on a Raman microscope (Renishaw inVia) with a 532 nm Ar laser.

### Electrochemical evaluation

4 mg of the synthesized MoS_2_ NS/MWCNT hybrid material and 80 μL of 5 wt % Nafion solution were dispersed in 1 mL of a water/ethanol mixture (3:1 v/v) followed by a sonication for 15 min to obtain a homogeneous catalytic slurry. Afterward, a glassy carbon electrode (GCE) with a diameter of 3 mm, which was polished by alumina suspensions, was treated with 5 mL of the catalytic slurry and dried at a temperature of 26 °C. In addition, pure MWCNTs, MoS_2_ NFs, and Pt/C modified electrodes were prepared by the same method for comparison purposes. The HER activities of these catalysts were evaluated via linear sweep voltammetry (LSV) in 0.5 M H_2_SO_4_ solution at a scan rate of 5 mV s^−1^ and at a temperature of 26 °C. LSV measurements were conducted using an electrochemical workstation (CHI 660D) and a standard three-electrode setup containing a saturated calomel electrode (SCE) as the reference electrode, a graphite rod as the counter electrode, and the modified GCEs as working electrodes. Before electrochemical measurements, the polarization curves were corrected for iR losses, the potentials were calibrated using a reversible hydrogen electrode (RHE), and the utilized electrolytes were degassed by bubbling Ar gas for 1 h. The AC impedance amplitude measured in the frequency range between 105 Hz and 101 Hz was below 5 mV.

### Data Availability

All data generated or analysed during this study are included in this published article (and its Supplementary Information files).

## Results and Discussion

**Figure 1:**
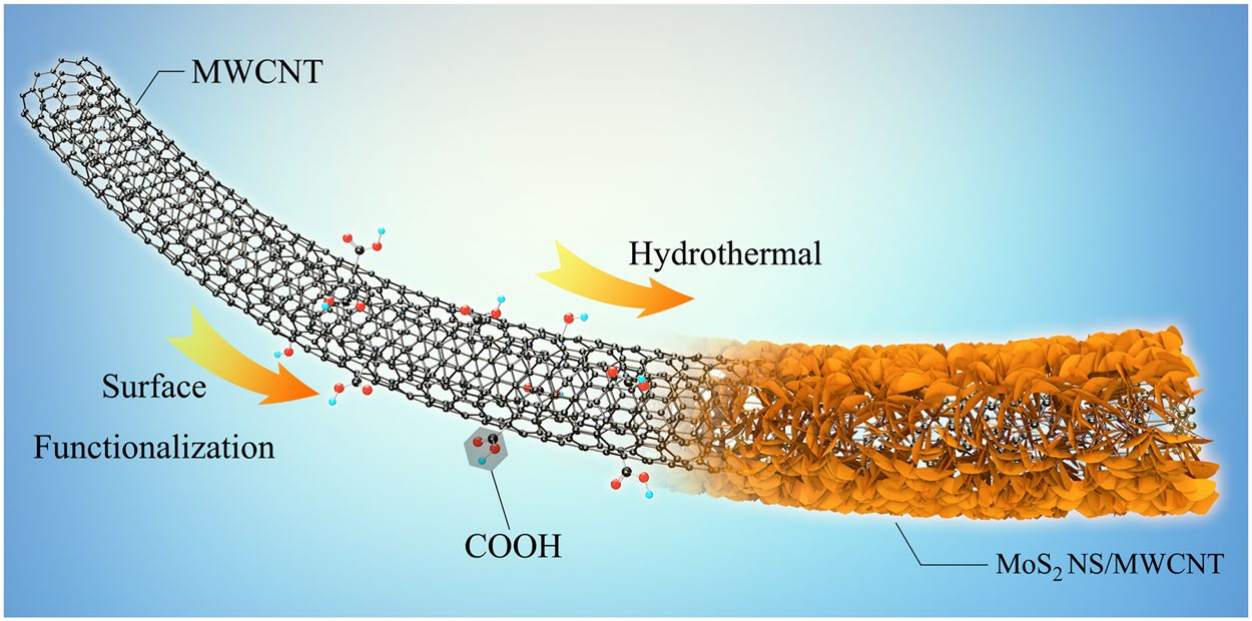
Schematic illustration of synthesis process for MoS_2_ NS/MWCNT hybrid.

As schematically illustrated in Fig. 1, a simple synthetic procedure was used to functionalize the MWCNT surface and thus improve its hydrophilicity. Afterward, specified amounts of sodium molybdate, thiourea, and surface-functionalized MWCNTs were mixed in DI water and heated to 180 °C for 24 h. (The details of the utilized synthesis procedure can be found in the Supplementary Information file.) The morphology and structure of the non-functionalized and functionalized MWCNTs were studied in detail by SEM and TEM, while their atomic oxygen concentrations were estimated via XPS. The obtained results revealed a slight increase in the oxygen content on the surface of functionalized MWCNTs, while their side walls contained no apparent defects (Table S1 and Figures S1–S4 in the Supplementary Information file). Further experimental studies confirmed that the hydrophilicity of the surface-functionalized MWCNTs was improved significantly (Figure S5 in the Supplementary Information file). Due to the high hydrophilicity of the functionalized MWCNTs, the latter was highly dispersed in the aqueous solution during the growth of ultrathin MoS_2_ NSs on their surface without stacking. This was proved by an additional experiment explained in the Supplementary Information file (see Figures S6 and S7). Peaks at approximately 381 and 407 cm^−1^ in the Raman spectrum of the MoS_2_ NSs/MWCNT hybrid are assigned to the in-plane ${{\rm{E}}}_{2{\rm{g}}}^{1}$ mode and the out-of-plane A_1g_ mode, respectively^38^. The ${{\rm{E}}}_{2{\rm{g}}}^{1}$ mode involved in-plane displacement of Mo and S atoms, while the A_1g_ mode represented out-of-plane symmetric displacements of S atoms (see Figure S8)^39^. In order to detect the presence of the MWCNT, we also measured the Raman spectra of samples in the region from 1100 to 1800 cm^−1^ (see Figure S8)^40^. Raman analysis confirms the layered structure of MoS_2_ and its attachment to the MWCNT.

**Figure 2:**
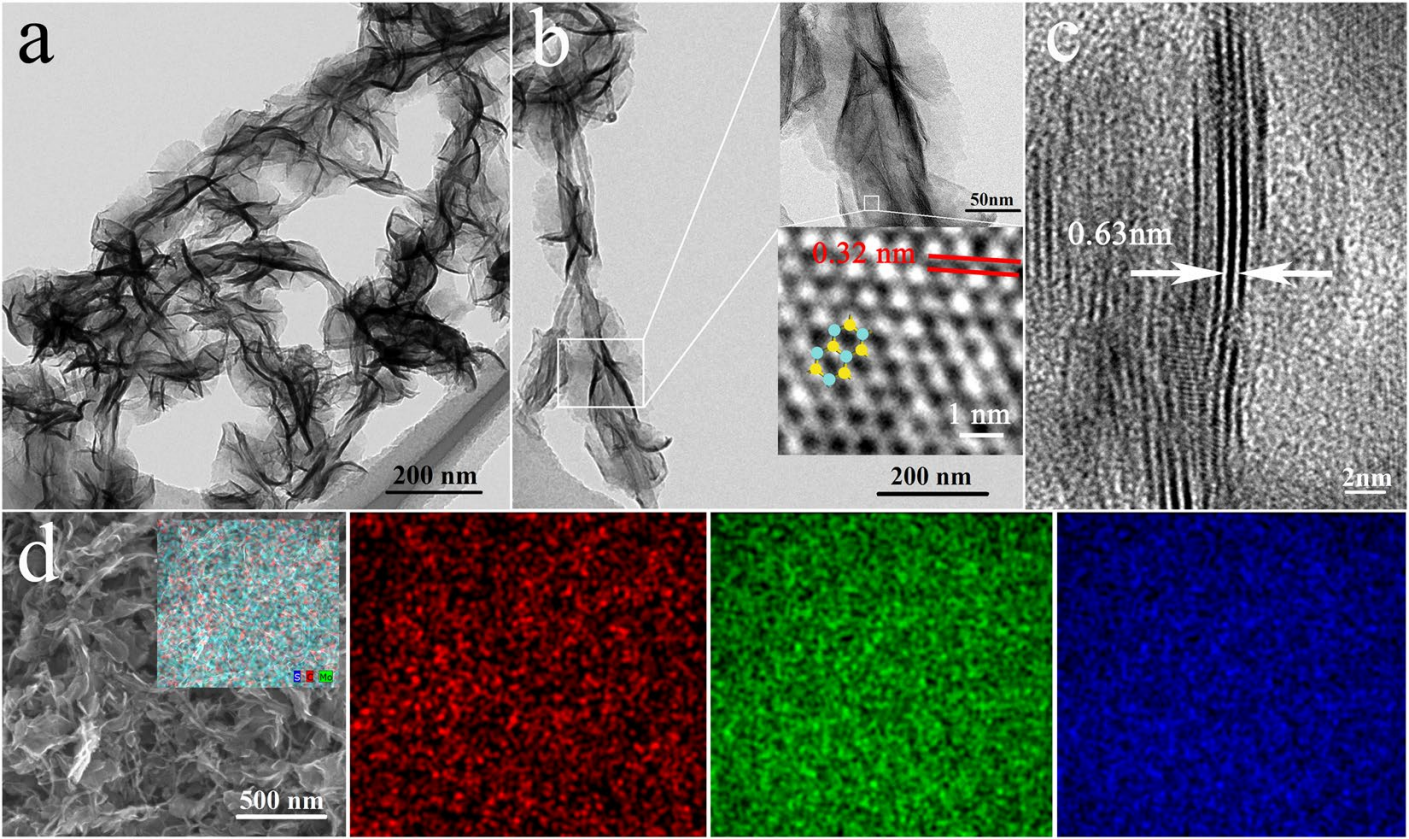
TEM image (**a**) and HRTEM images (**b**,**c**) of the MoS_2_ NS/MWCNT hybrid (the insert graph is a blow-up image of Mo (the yellow dot) and S (the blue dot) atoms and their honeycomb arrangement). (**d**) SEM image and the corresponding EDS elemental mappings of C, Mo, and S for the MoS_2_ NS/MWCNT hybrid.

The hierarchical architecture, morphology, and lattice structure of the as-prepared MoS_2_ NS/MWCNT composite were investigated by TEM and SEM. Figure 2a and d contain the TEM image and the SEM image of the uniform petal-like MoS_2_ NSs grown on the outer MWCNTs surface with diameters of about 150 nm, which show that each MoS_2_ NS is composed of 3–8 MoS_2_ layers with an interlayer spacing of 0.63 nm and particular lattice structure (Fig. 2b and c). The HRTEM image shown in the insert graphs of Fig. 2b clearly reveal the honeycomb structure of MoS_2_ where the Mo and S sites can be identified by differences in their contrast^41^. In addition, the lattice constant of MoS_2_ measured from HRTEM image is about 0.32 nm which agree with the previously reported value^42^. The results of SEM image and the corresponding EDS mapping of the C, Mo, and S elements (Fig. 2d) revealed that the produced MoS_2_ NSs were uniformly distributed on the MWCNTs substrate. In previous studies, DMF solvent was used to prepare hybrid nano-sized MoS_2_ structures grown on carbon-based substrates since the use of water as solvent generated a mixture of aggregated MoS_2_ species and carbon-based materials^28, 31^. As indicated by the results obtained in this study, petal-like MoS_2_ NSs can be uniformly grown on MWCNTs in an aqueous solution using a simple, environmentally friendly, and inexpensive hydrothermal method. Due to the uniform distribution of the produced MoS_2_ NSs, they contained a larger number of catalytically active edge sites, which could be attributed to the relatively mild synthesis conditions. The latter included (a) a relatively low temperature of 180 °C, (b) conductive MWCNTs with large outer diameters, and (c) low concentrations of the MWCNT, thiourea, and sodium molybdate components. As a result, the concentration of MoO_4_^−2^ anions in the reaction solution was very low, while the large outer diameter of MWCNTs ensured selective, slow, and uniform growth of Mo-containing species on the MWCNT surface.

Moreover, two additional experiments were performed to repeat the hydrothermal synthesis procedure described in previous studies using water as a solvent. In one experiment, the amount of thiourea and sodium molybdate were increased, while in the other experiment, single-wall CNTs with smaller inner/outer diameters were utilized as composite substrates (the details of these experiments can be found in the Supplementary Information file). As a result, two different mixtures containing conglobate MoS_2_ particles and aggregated MoS_2_ particles connected via CNT wires, respectively, were obtained, which was in good agreement with the data reported earlier (see Figures S9 and S10 in the Supplementary Information file)^29, 31^.

**Figure 3:**
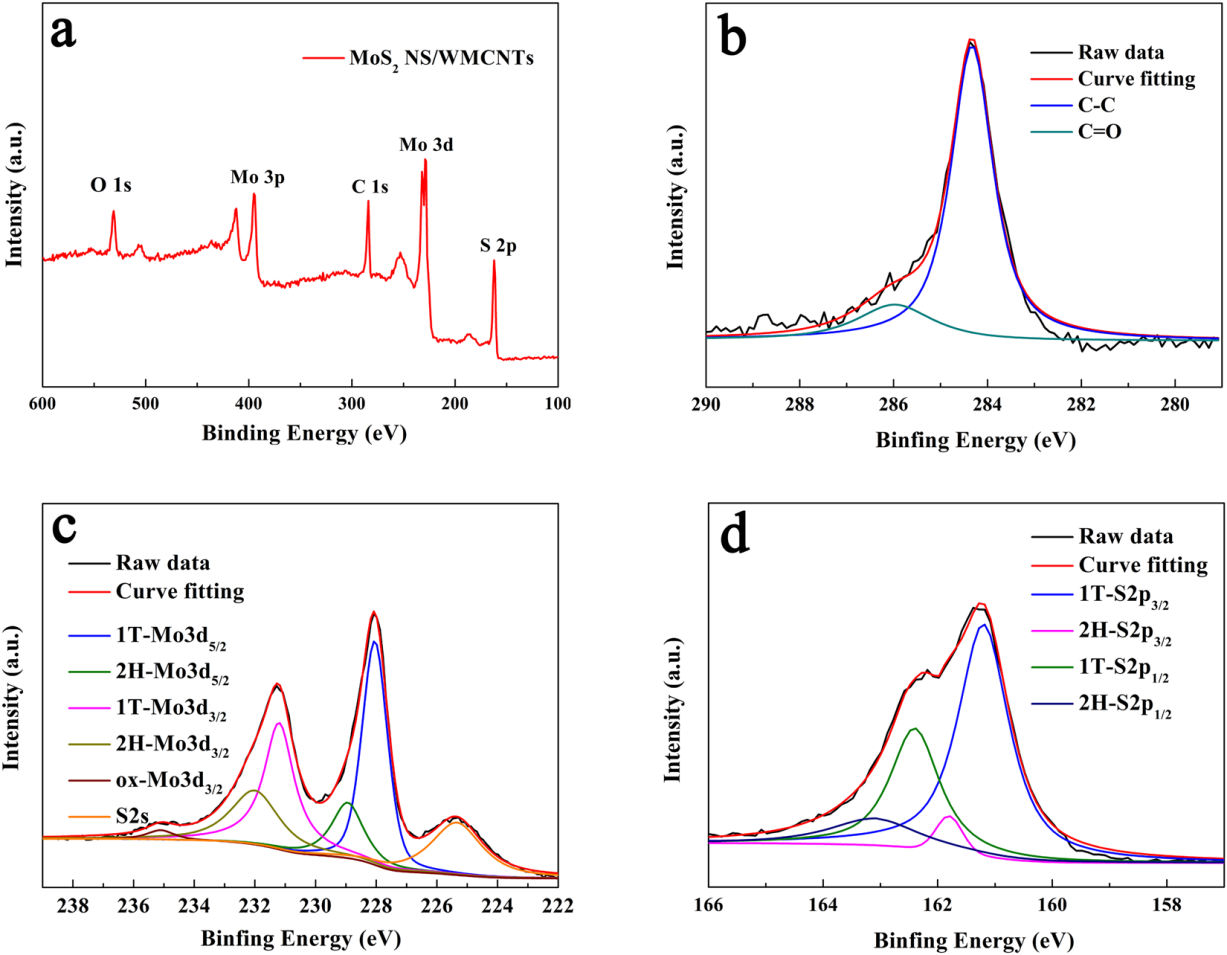
(**a**) XPS survey spectra and (**b**–**d**) high-resolution XPS spectra of the MoS_2_ NS/MWCNT hybrid.

X-ray photoelectron spectroscopy (XPS) spectra were recorded to gain further insights into the chemical nature and bonding state of the MoS_2_ NSs deposited on the MWCNTs surface. The peaks depicted in Fig. 3a confirmed that the main elements of the prepared MoS_2_ NS/MWCNT hybrid material were Mo, S, C, and O. Figure 3b contains the high-resolution C 1 s XPS spectrum, which was subsequently fitted with two different components. The main peak centered at 284.6 eV represents a standard C peak. The peak at around 285.9 eV indicates the presence of C atoms bound to oxygen atoms, which originated from the nitric acid-treated MWCNT surface with a small number of oxygen-containing functional groups^43^. Figure 3c and d showed the XPS spectra of the Mo 3d, S 2 s, and S 2p regions. The Mo 3d spectra consist of weak peaks at around 229 and 232 eV that correspond to Mo^4+^ 3d_5/2_ and Mo^4+^ 3d_3/2_ components of the 2 H phase of MoS_2_, respectively. Deconvolution of these peaks reveals additional strong peaks that are shifted to lower binding energies by ~0.9 eV with respect to the position of the 2H-Mo3d peaks and they arise from the 1T phase^44^. Equally, in the S2p region of the spectra, additional strong peaks (1T-S2p) are found beside the known weak peaks of 2H-S2p_1/2_ and 2H-S2p_3/2_ of MoS_2_, which appear at 163 and 161.9 eV, respectively^45^. Furthermore, the relatively weak peak detected at 235.1 eV corresponds to the Mo^6+^ oxidation state (The latter feature most likely resulted from the formation of a very small amount of MoO_3_ species during catalyst preparation). These results show that the prepared MoS_2_ NS/MWCNT hybrid material with a very high concentration of metallic 1T-MoS_2_^46, 47^.

**Figure 4:**
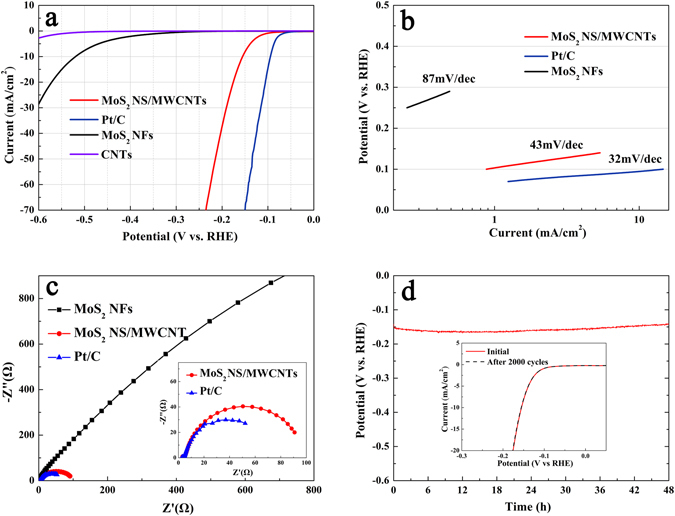
Polarization curves for catalysts (**a**) and the corresponding Tafel plots (**b**). (**c**) Impedance spectroscopy at an overpotential of 155 mV. (**d**) Durability test for the MoS_2_ NS/MWCNT hybrid catalyst.

The catalytic HER performance of the prepared MoS_2_ NS/MWCNT nanohybrid was evaluated at a temperature of 26 °C using the described three-electrode setup. As a reference, the electrocatalytic activity of the commercial Pt/C catalyst (containing 20 wt % Pt on Vulcan carbon black) was investigated as well. Different polarization curves obtained for the studied catalysts via LSV are shown in Fig. 4a. It was found that the Pt/C catalyst exhibited very strong HER performance with an onset potential close to zero, while the MoS_2_ NS/MWCNT hybrid material was characterized by a small onset potential of about 50 mV. In a sharp contrast, both the pure MWCNTs and MoS_2_ NFs synthesized by the hydrothermal method (the detailed structural characteristics of the produced MoS_2_ NFs are shown in Figure S11 in the Supplementary Information file) exhibited either the complete absence or very poor HER electrocatalytic activity due to the low current density and large onset potential. At 10 mA cm^−2^, the applied overpotential of the MoS_2_ NS/MWCNT hybrid material is approximately 155 mV, substantially lower than that of the MoS_2_ NFs (ca. 520 mV) and the rGO. The linear segments of the corresponding Tafel plots (Fig. 4b) were fit with the Tafel equation *η* = *b* × log *j* + *a*, where *j* was the current density, and *b* was the Tafel slope. As a result, the Tafel slopes of 87, 43, and 32 mV/decade were obtained for MoS_2_ NFs, MoS_2_ NS/MWCNT, and Pt/C, respectively. Furthermore, the results of several additional experiments showed that the addition of 56 mg of sodium molybdate, 67 mg of thiourea, and 11 mg of surface-functionalized MWCNTs into 70 mL of DI water at a temperature of 180 °C led to the successful formation of the high-performance MoS_2_ NS/MWCNT HER catalyst (Table S2 and Figures S12–S15 in the Supplementary Information file). The superior catalytic activity, which substantially improves the charge transfer kinetics of HER can be attributed to the strong electronic coupling between the MWCNTs and MoS_2_ NSs and its also attributed to the very high concentration of metallic 1T phase of the MoS_2_ NSs^48^.

To maximize this effect, impedance measurements were performed at an applied overpotential of *η* = 155 mV. As shown in Fig. 4c, the same amount of the MoS_2_ NS/MWCNT catalyst exhibited a lower alternating current impedance of around 100 Ω, which was very close to that of the Pt/C catalyst (around 70 Ω) and was much lower than that of the MoS_2_ NFs (around 10 kΩ). Another important characteristic of an electrocatalyst with superior properties is high durability. To further evaluate the long-term stability of the synthesized MoS_2_ NS/MWCNT catalyst in an acidic environment and under a cathodic current of 10 mA cm^−2^, there is no noticeable degradation over a 48 h galvanostatic test, which indicates an excellent electrochemical HER stability (Fig. 4d). In addition, it was exposed to 2000 continuous treatment cycles. The obtained I*-*V curves were very similar to those recorded previously and exhibited negligible losses of the cathodic current (the insert of Fig. 4d). The TEM images depicted in Figure S16 (in the Supplementary Information file) showed that the original morphology of the hybrid catalyst was well preserved after acidic treatment.

Remarkably, Tafel slopes are one of the most significant factors that can help to elucidate the HER mechanism. According to the classic theory^49–51^, the Tafel slopes estimated for typical Volmer, Heyrovsky, and Tafel reactions were around 120 mV dec^−1^, 40 mV dec^−1^, and 30 mV dec^−1^, respectively (1–3). The following reactions describe the HER steps in acidic aqueous media, where CH_ads_ denotes the hydrogen atoms chemically adsorbed on the catalyst (C) active sites. Since the Tafel slope obtained for the MoS_2_ NS/MWCNT hybrid catalyst in this work was equal to 43 mV dec^−1^, the related HER process consisted of a combination of the Volmer reaction (involving an electrochemical desorption step that converts protons into absorbed hydrogen atoms on the catalyst surface), and the Heyrovsky reaction (involving the formation of surface hydrogen molecules). In other words, the rate-determining step corresponds to the electrochemical desorption of H_ads_ and H_3_O^+^ species, during which hydrogen molecules are formed, and the entire HER proceeds through the Volmer-Heyrovsky mechanism.

1 $${{\rm{H}}}_{3}{{\rm{O}}}^{+}+{{\rm{e}}}^{-}+{\rm{C}}\to {{\rm{CH}}}_{{\rm{ads}}}+{{\rm{H}}}_{2}{\rm{O}}$$

2 $${{\rm{H}}}_{3}{{\rm{O}}}^{+}+{{\rm{e}}}^{-}+{{\rm{CH}}}_{{\rm{ads}}}\to {\rm{C}}+{{\rm{H}}}_{2}+{{\rm{H}}}_{2}{\rm{O}}$$

3 $${{\rm{CH}}}_{{\rm{ads}}}+{{\rm{CH}}}_{{\rm{ads}}}\to 2{\rm{C}}+{{\rm{H}}}_{2}$$

**Figure 5:**
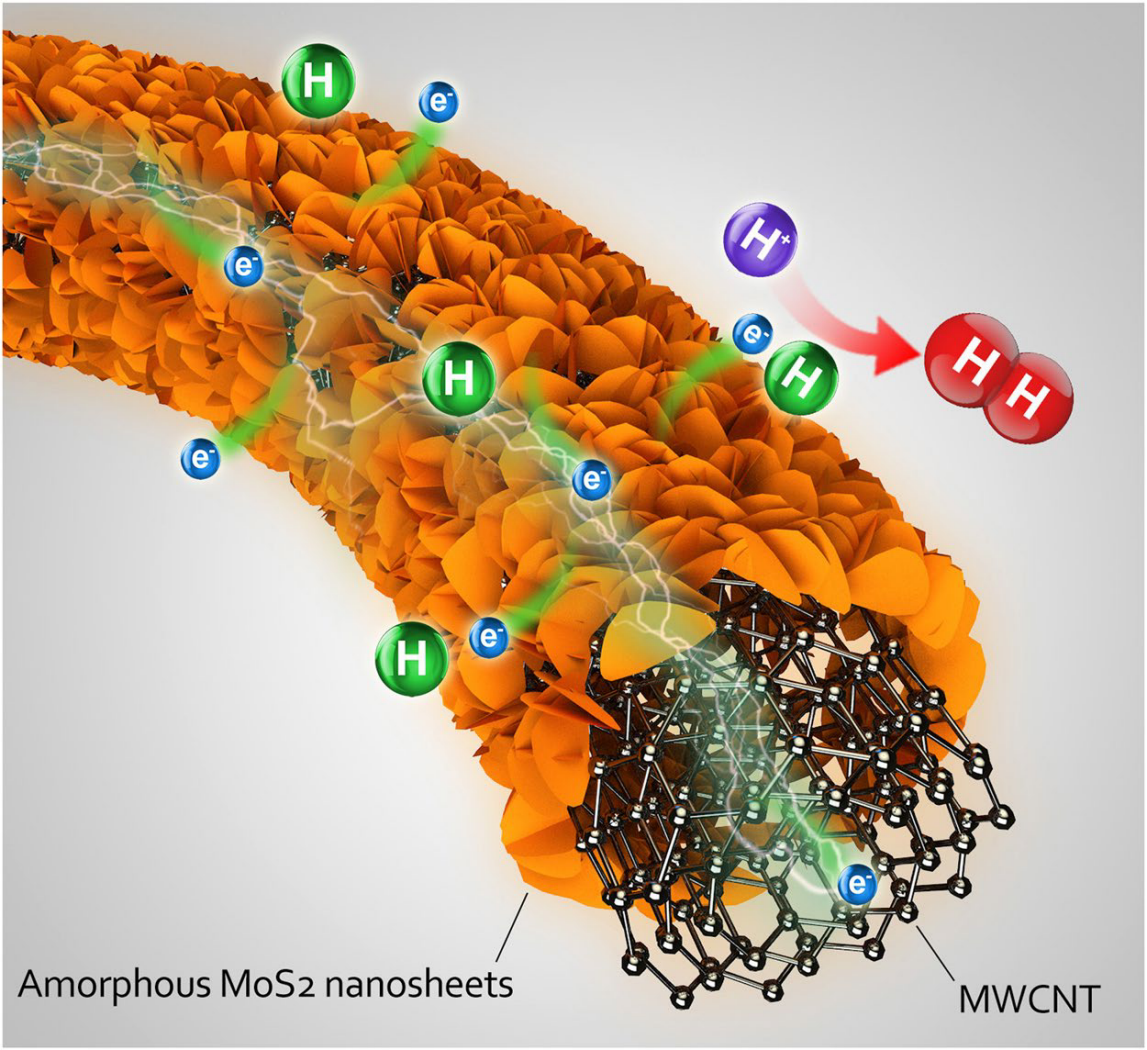
Schematic illustration of the mechanism governing the electrocatalytic HER on the MoS_2_ NS/MWCNT structure.

To elucidate the synergistic effect produced by the synthesized MoS_2_ NS/MWCNT hybrid material on the catalytic process in more detail, a simple model (Fig. 5) can be considered. The obtained hybrid contains a large number of active HER catalytic sites due to the abundance of accessible edges resulting from the small sizes and high dispersion of MoS_2_ NSs on the MWCNT surface. The use of MWCNTs contributes to the rapid electron transport from the electrode to the substrate and then to the synthesized metallic 1T phase MoS_2_ NSs. Therefore, the produced MoS_2_ NS/MWCNT composite can effectively reduce dissociated H^+^ ions and release H_2_ molecules on a large number of active sites.

## Conclusion

In summary, a nanohybrid catalyst for the HER that contained MoS_2_ NSs uniformly grown on MWCNTs was fabricated by a facile hydrothermal approach. In addition, it was successfully demonstrated that water could be used as the reaction solvent. Owing to the excellent electrical coupling between the petal-like MoS_2_ NSs and the underlying MWCNTs surface, the produced MoS_2_ NS/MWCNT hybrid catalyst exhibited excellent HER catalytic properties corresponding to a low overpotential, small Tafel slope, and long cycle life. Therefore, this work describes an environmentally friendly and inexpensive method for the efficient fabrication of the MoS_2_ NS/MWCNT hybrid catalyst for the HER.

## Electronic supplementary material

Supplementary Information
