# Supplementary material for: A Clean and Facile Synthesis Strategy of MoS2 Nanosheets Grown on Multi-Wall CNTs for Enhanced Hydrogen Evolution Reaction Performance
Source: Sci Rep. 2017 Aug 18;7:8825. doi: 10.1038/s41598-017-09047-x (PMC5562701; doi:10.1038/s41598-017-09047-x)
Supplement: Supplementary file 1 — Supplementary Information [file 41598_2017_9047_MOESM1_ESM.docx]

A Clean and Facile Synthesis Strategy of MoS_2_ Nanosheets Grown on Multi-Wall CNTs for Enhanced Hydrogen Evolution Reaction Performance

JiamuCao^1^, Jing Zhou^1^, Yufeng Zhang^1,2, *^, Xiaowei Liu^1,2^

^1^MEMS Center, Harbin Institute of Technology, Harbin, 150001, PR China.

^2^Key Laboratory of Micro-systems and Micro-structures Manufacturing, Ministry of Education, Harbin, 150001, PR China.

^*^Corresponding author. E-mail adress: [yufeng_zhang@hit.edu.cn](mailto:yufeng_zhang@hit.edu.cn) (Y. F. Zhang)

**Supplementary Information**

**Surface functionalization of MWCNTs**

MWCNTs with average outer/inner diameters of 15 nm/8 nm and lengths of 50 μm were purchased from XF Nano, Ltd (Nanjing, CN). To improve the hydrophilicity of the nanotube substrate, the MWCNT surface was functionalized prior to MoS_2_ deposition. First, 100 mg of MWCNTs was sonicated in 500 mL of concentrated HNO_3_ solution (30-70% w/w) at a temperature of 60 °C for 1 h. After that, the MWCNT sample was washed with deionized (DI) water and dried at 60 °C in a vacuum oven for 6 h.

**Preparation of MoS_2_** **NSs/MWCNTs hybrid catalyst**

During the synthesis of the MoS_2_ NS/MWCNT hybrid material, 56 mg of sodium molybdate, 67 mg of thiourea, and 11 mg of surface-functionalized MWCNTs were added to 70 mL of DI water and sonicated for 1 h was transferred to a 100-mL Teflon-lined autoclave and heated at 180 °C for 24 h. The resulting dark suspension was collected via centrifugation at a speed of 6000 rpm, washed with DI water and ethanol, and dried in the vacuum oven at 60 °C. For comparison purposes, MoS_2_ nanoflowers (NFs) were synthesized via a similar method without MWCNT addition.

**Additional experiment**

Two additional experiments were performed to repeat the synthesis in the previous reports via hydrothermal method using water as a solution. In one experiment, 151 mg sodium molybdate, 200 mg thiourea, and 10 mg CNTs were added in 70 mL DI water and sonicated for 30 min. The mixture was then transferred to a Teflon-lined autoclave and heated at 250 °C for 24 h. The dark suspension was filtered and washed with DI water for several times before dried in vacuum oven [1]. In another experiment, 15 mg sodium molybdate, 18 mg thiourea, and 4 mg of the surface functionalized MWCNTs, were dissolved in 15 mL DI water. Then the mixture was well dispersed by sonication for 30 min, transferred into an autoclave, and reacted at 210 °C for 18 h. The resulting products were collected by centrifugation and subsequently washed with DI water and anhydrous ethanol several times. The final products were obtained after drying at 60 °C for 24 h [2].

**Table S1.** The test details of XPS

| **Type** | **Nane** | **Start Binding Energy**  **(eV)** | **Peak Binding Energy**  **(eV)** | **End Binding Energy**  **(eV)** | **At.%** |
| --- | --- | --- | --- | --- | --- |
| MWCNTs | C1s | 298.38 | 284.64 | 279.58 | 98.24 |
|  | O1s | 545.38 | 531.97 | 525.58 | 1.76 |
| Surface Functionalized MWCNTs | C1s | 298.38 | 284.57 | 279.58 | 87.87 |
|  | O1s | 545.38 | 532.08 | 525.58 | 12.13 |

**Table S2.** Performance comparison of HER catalyst samples

| No. | Carbon source  (mg) | Sodium molybdate  (mg) | Thiourea  (mg) | DI water  (ml) | Temperature  °C | Time  (h) | applied overpotential  (mV) | Tafel slope  (mV dec^-1^) |
| --- | --- | --- | --- | --- | --- | --- | --- | --- |
| 1 | WMCNTs  11 mg | 56 mg | 67 mg | 70 ml | 180 °C | 24 h | 155 mV | 43 mV dec^-1^ |
| 3 | None | 56 mg | 67 mg | 70 ml | 180 °C | 24 h | 520 mV | 87 mV dec^-1^ |
| 4 | WMCNTs  11 mg | 56 mg | 67 mg | 70 ml | 250 °C | 18 h | 295 mV | 69 mV dec^-1^ |
| 5 | WMCNTs  11 mg | 28 mg | 34 mg | 70 ml | 180 °C | 24 h | 315 mV | 71 mV dec^-1^ |
| 6 | CNTs  10 mg | 151 mg | 200 mg | 70 ml | 250 °C | 24 h | 455 mV | 80 mV dec^-1^ |
| 7 | CNTs  4 mg | 15 mg | 18mg | 15 ml | 210 °C | 18 h | 286 mV | 66 mV dec^-1^ |

① No.2 is commercial Pt/C catalyst (20 wt% Pt on Vulcan carbon black).

② The table’s color is corresponding to the line`s color in Figure S14 and Figure S15.


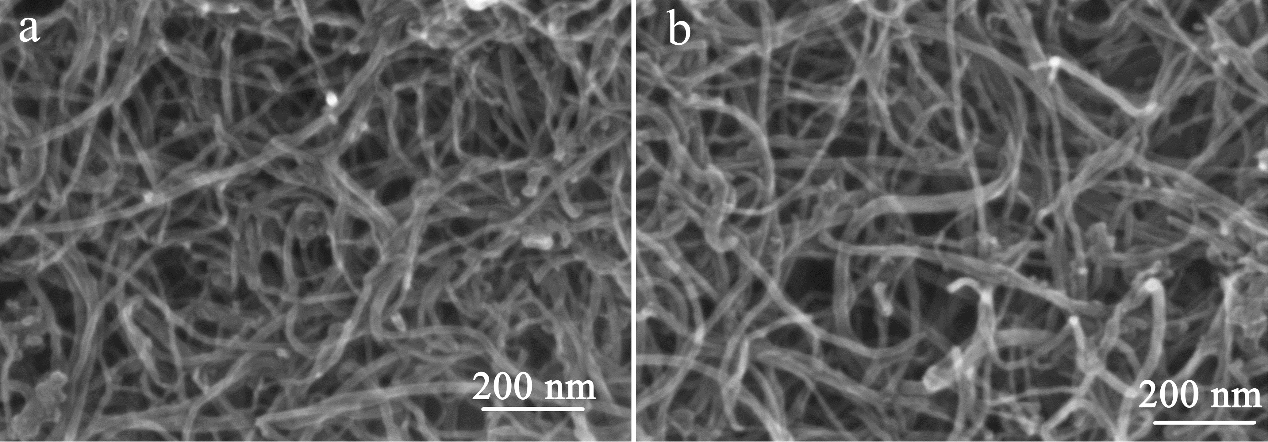


**Figure S1.** SEM images of MWCNTs (a) and surface functionalized MWCNTs (b).


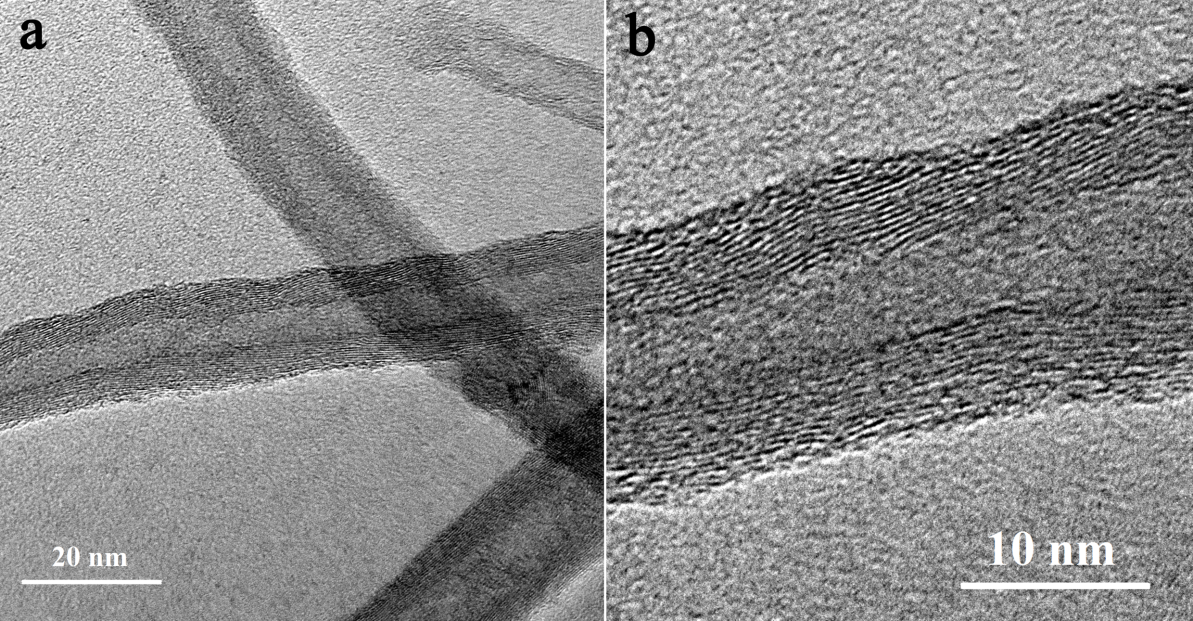


**Figure S2.** TEM image of MWCNTs (a) and surface functionalized MWCNTs (b).


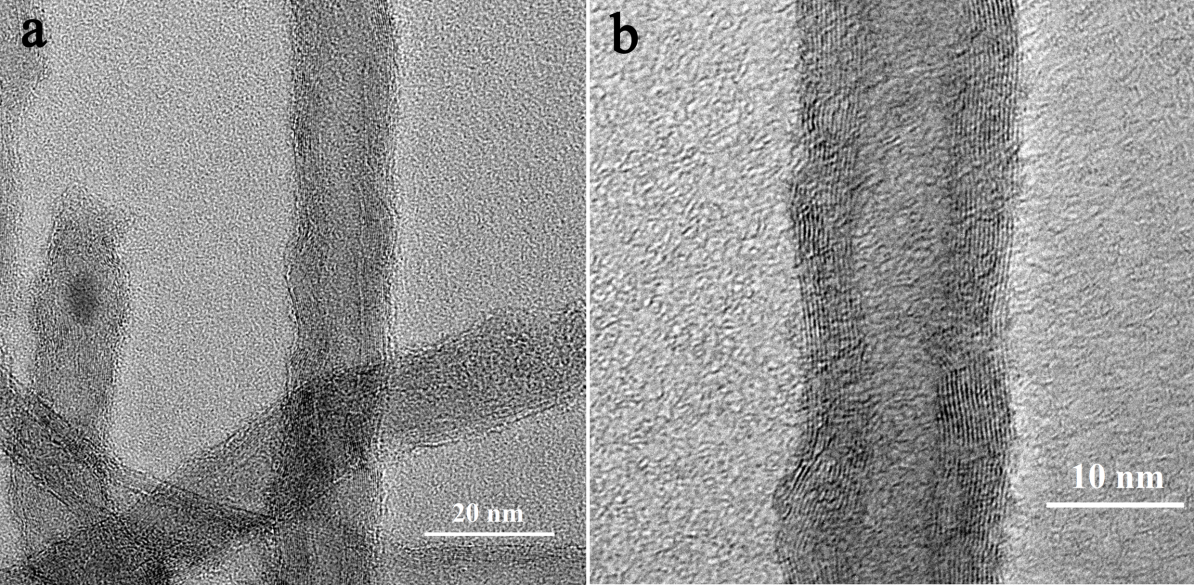


**Figure S3.** TEM image of surface functionalized MWCNTs (a) and HRTEM image of surface functionalized MWCNTs (b).

**
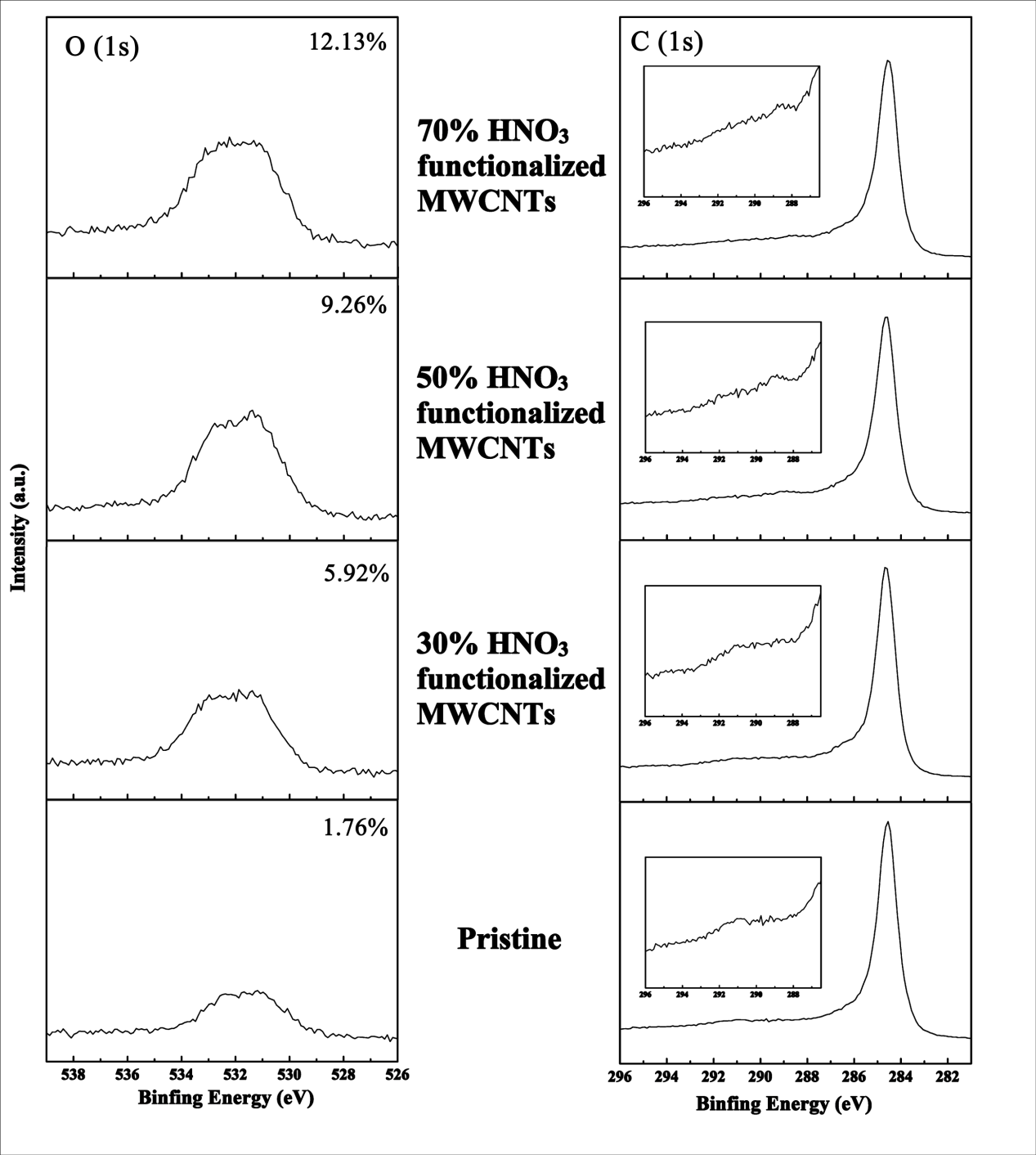
**

**Figure S4.** C (1s) and O (1s) XPS regions of surface functionalized MWCNTs and MWCNTs. It should be noted that the O (1s) peaks have been normalized to the accompanying C (1s) peak areas in each case. The insert in the C (1s) region shows the π–π* transition at ≈291 eV.

The easiest and most reliable way to control the extent of the functionalized MWCNTs was to vary the oxidant concentration, specifically the w/w% HNO_3_ (from 0–70%) [3, 4]. Using XPS analysis, the increase in oxidation as the w/w% HNO_3_ increases is clearly observed by comparing changes in the intensity of the O (1s) spectral envelope (Figure S4). The insert graphs in Figure S4 also show that the π–π* shake-up transition, associated with the delocalized π-electrons in MWCNTs, is attenuated as the level of oxidation increases. The results are consistent with previous reports [3]. Further experimental studies confirmed that the hydrophilicity of the surface-functionalized MWCNTs was improved significantly (Figure S4 and S5).


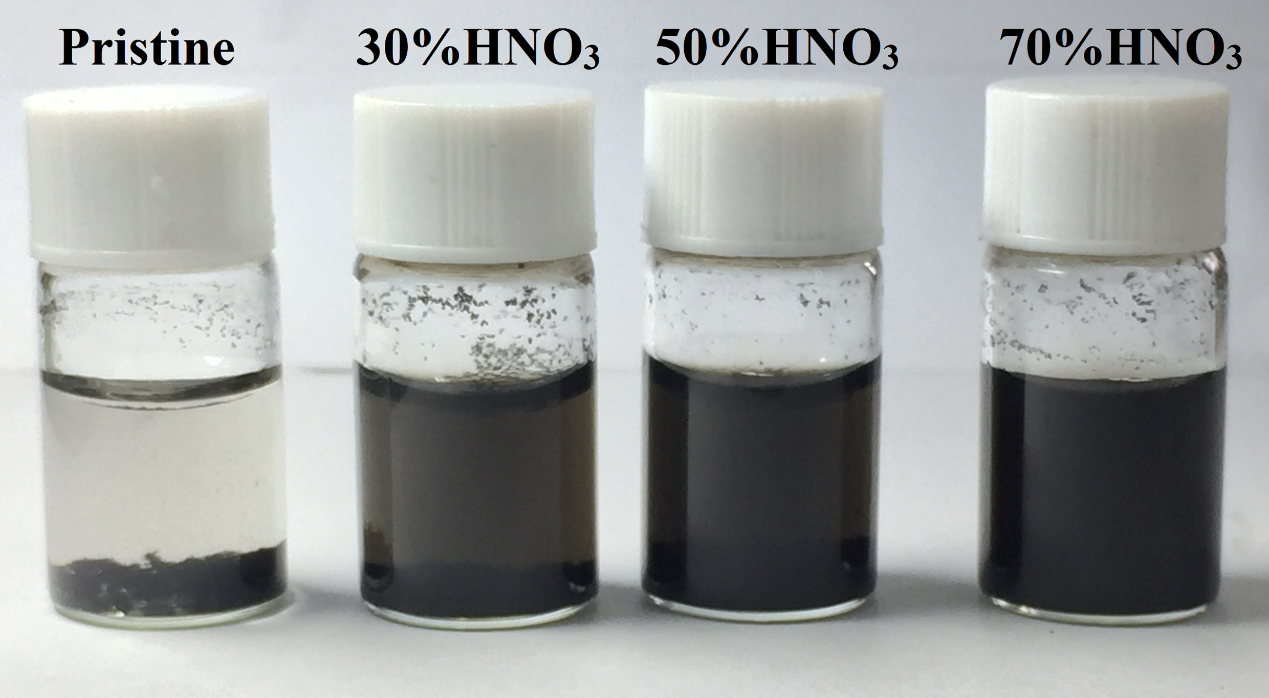


**Figure S5.** Hydrophilicity test of surface functionalized MWCNTs and non-functionalized MWCNTs.


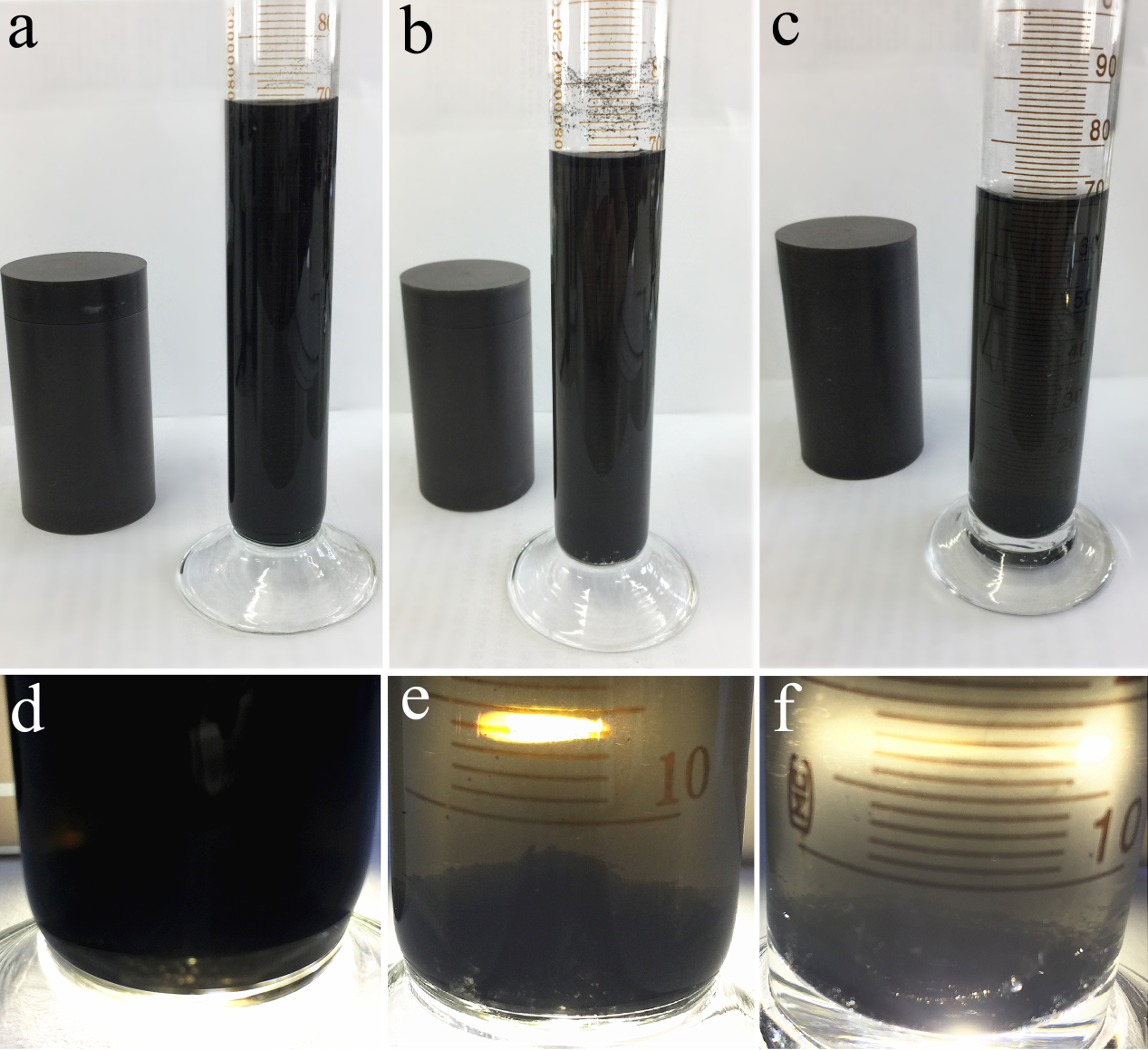


**Figure S6. (**a) and (d) are the surface-functionalized MWCNTs solution, (b) and (d) are the MWCNTs solution, (c) and (f) are the graphene solution.


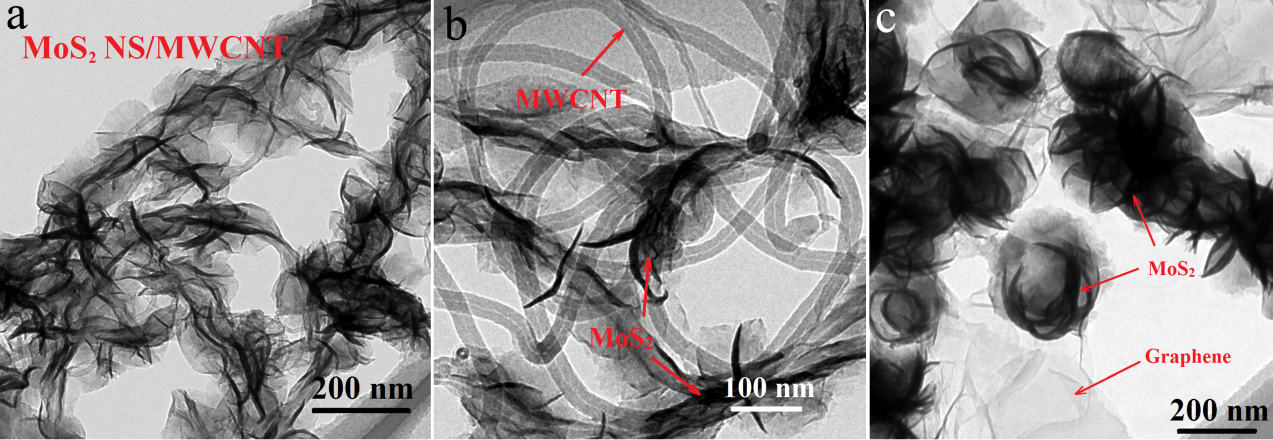


**Figure S7.** TEM images of (a)MoS_2_ NS/MWCNT hybrid, (b) the mixture of MoS_2_ and MWCNT, and (c) the mixture of MoS_2_ and graphene.

A set of additional tests proved that due to the high hydrophilicity of the functionalized MWCNTs, the latter were highly dispersed in the aqueous solution during the growth of ultrathin MoS_2_ NSs on their surface without stacking.

56 mg of sodium molybdate, 67 mg of thiourea, and 11 mg of carbon source (surface-functionalized MWCNTs, MWCNTs and graphene) added to 70 mL of DI water and sonicated for 1 h was transferred to a 100 mL Teflon-lined autoclave and heated at 180 °C for 24 h. It can be seen that the surface-functionalized MWCNTs were high dispersive in water, while there are clear precipitation in the MWCNTs solution and the graphene solution (Figure S6). The resulting dark suspension was collected via centrifugation at a speed of 6000 rpm, washed with DI water and ethanol, and dried in the vacuum oven at 60 °C. TEM analysis have been found that using the surface-functionalized MWCNTs as carbon source can uniformly grow of ultrathin MoS_2_ NSs on their surface without stacking while using the MWCNTs and the graphene as carbon source generated the mixture of MoS_2_ and carbon source (Figure S7).


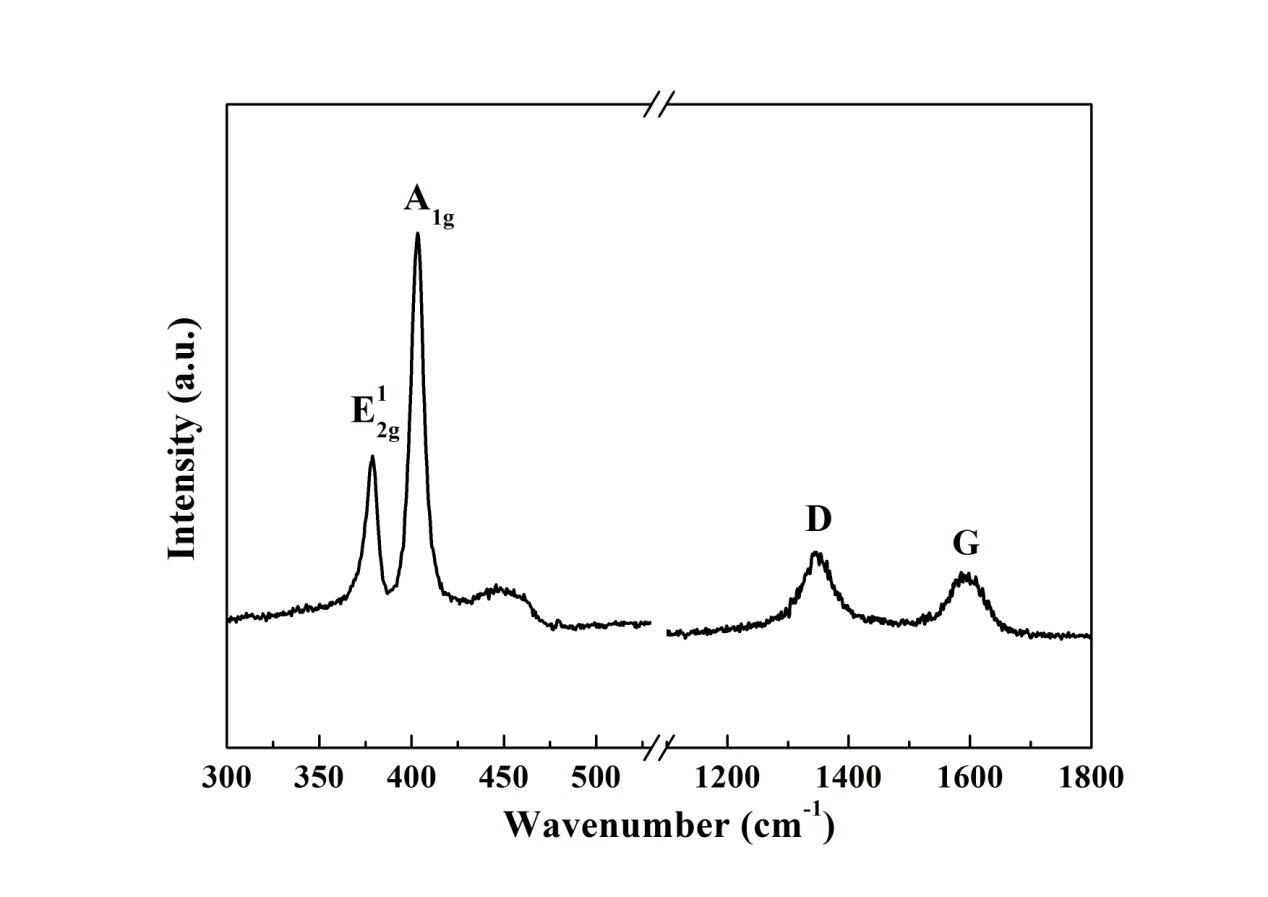


**Figure S8.** Raman spectra of MoS_2_ NS/MWCNT composite


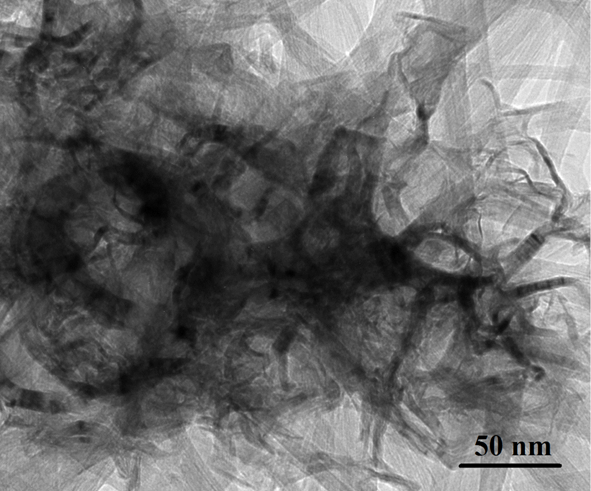


**Figure S9.** TEM image of the amount increased thiourea and sodium molybdate. The date is in the Table S2 (No. 6).


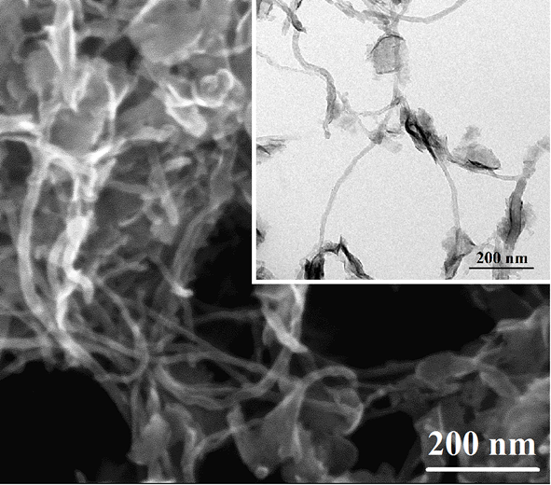


**Figure S10.** SEM image and insert TEM image of the single-wall CNTs substrate with smaller inner/outer diameter. The date is in the Table S2 (No. 7).

**
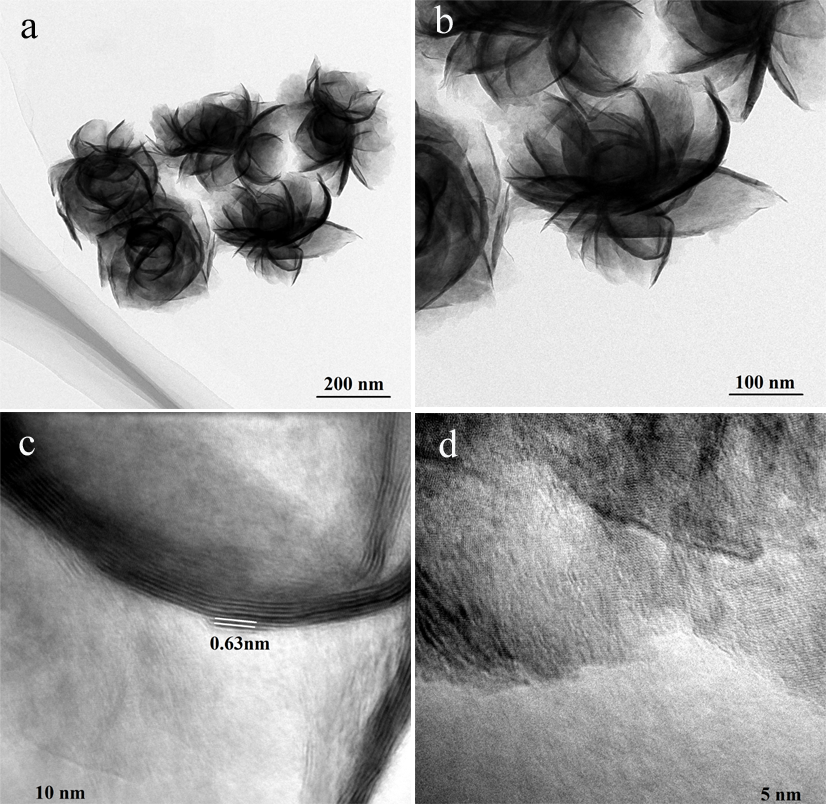
**

**Figure S11.** TEM image of MoS_2_ nanoflowers (NFs). The date is in the Table S2 (No. 3). It can be observed that each MoS_2_ NFs are composed of a few MoS_2_ layers with an interlayer spacing of 0.63 nm and the lattice structure also indicated.


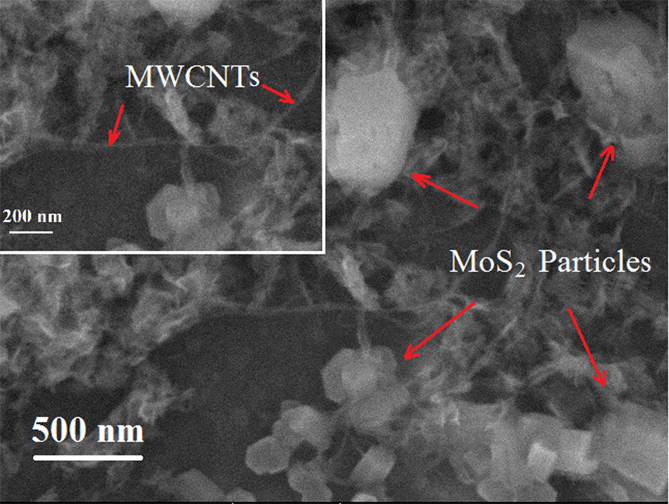


**Figure S12.** SEM image of the hybrid prepared at 250℃. The date is in the Table S2(No. 4). It can be clearly seen that the mixture contained a lot of aggregated MoS_2_ particles and bare WMCNTs, which on account of the high temperature made synthesis process too severe.


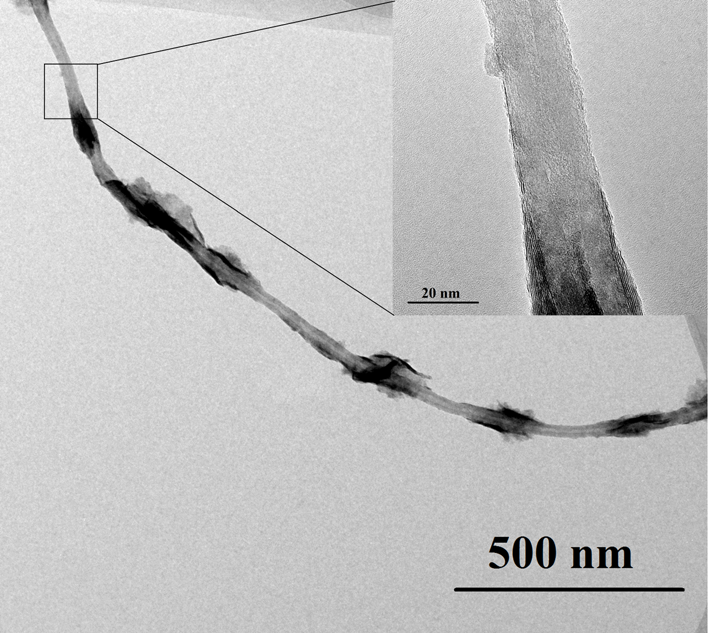


**Figure S13.** TEM image of the sample in the Table S2(No. 5). It can be seen that low amount of sodium molybdate and thiourea resulting inadequate synthesis of the materials.


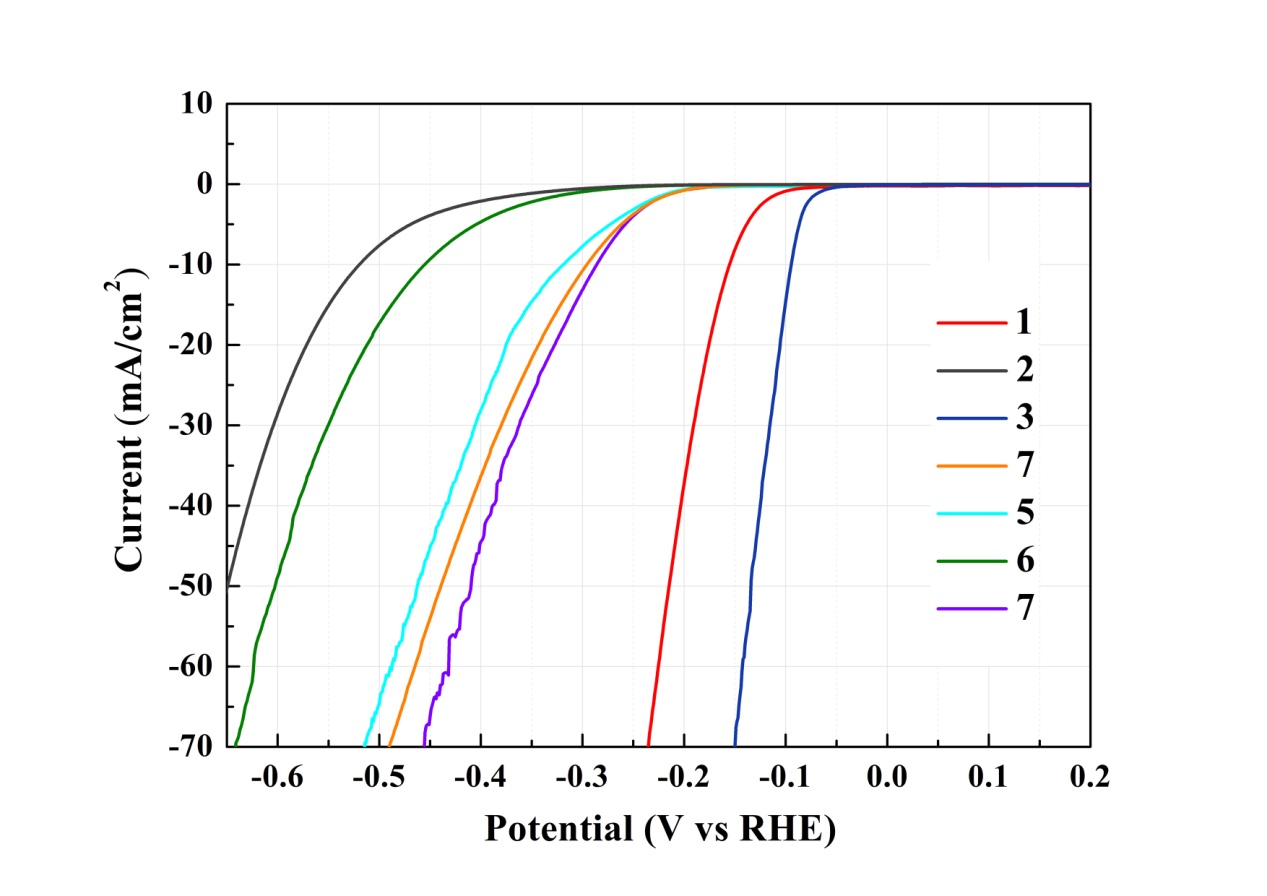


**Figure S14.** Polarization curves of the samples. The figure investigates that the sample possesses the lowest overpotential and the largest current densities.


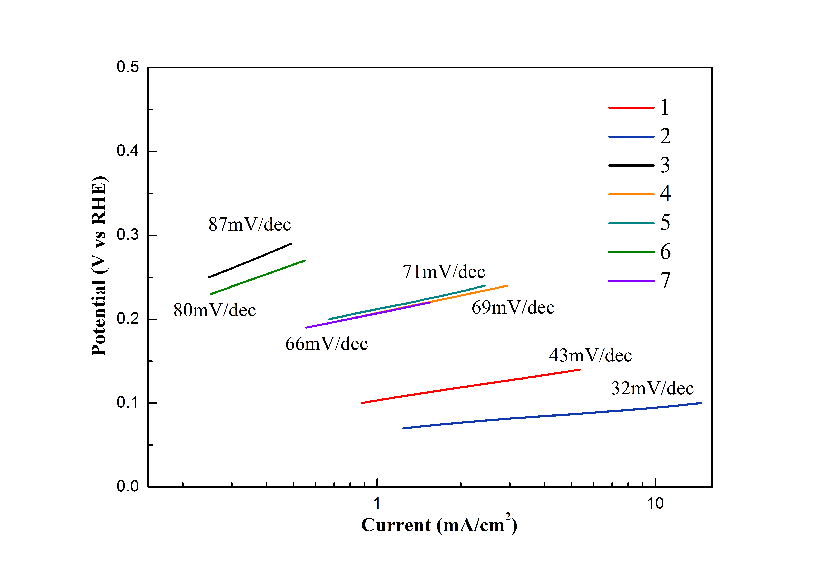


**Figure S15.** Tafel plots of the samples. The figure investigates that the sample exhibits a low Tafel slope which approximates to the Pt/C catalyst.

As a consequent, the supporting information revealed the key factor that successful synthesis of the high-performance MoS_2_ NS/WMCNTs catalyst in the water solution is based on appropriate substrate and the suitable amount of WMCNTs, sodium molybdate and thiourea.

**
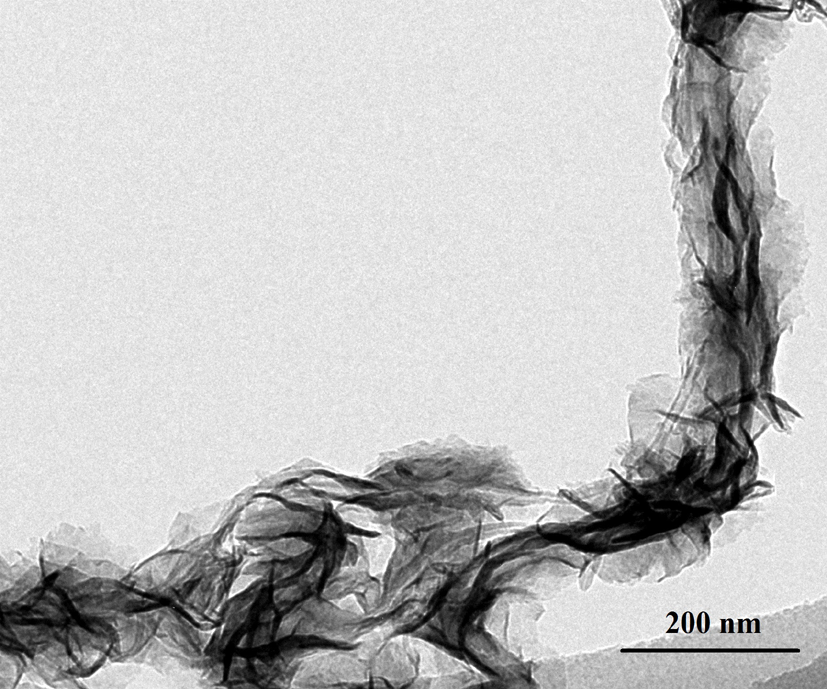
**

**Figure S16.** The TEM image of MoS_2_ NS/MWCNT catalyst exposed to 2000 continuous treatment cycles in an acidic environment. It depicted that the original morphology of the hybrid catalyst was well preserved after acidic treatment.

**Reference**

1. Li, J. Y. *et al.* A three-dimensionally interconnected carbon nanotube/layered MoS_2_ nanohybrid network for lithium ion battery anode with superior rate capacity and long-cycle-life. *Nano Energy.* **16,** 10-18 (2015).
2. Yan, Y. *et al.* Facile synthesis of low crystalline MoS_2_ nanosheet-coated CNTs for enhanced hydrogen evolution reaction. *Nanoscale.* **5,** 7768-7771 (2013).
3. Wepasnick, K. A. *et al.* Surface and structural characterization of multi-walled carbon nanotubes following different oxidative treatments. *Carbon.* **49,** 24-36 (2011).
4. Aviles F. *et al.* Evaluation of mild acid oxidation treatments for MWCNT functionalization. *Carbon*. **47**, 2970-2975 (2009).
